# Quality and reliability of osteoarthritis-related health information on short video platforms: a cross-sectional comparative study of TikTok and Bilibili

**DOI:** 10.3389/fdgth.2025.1623247

**Published:** 2025-11-07

**Authors:** Qi-Heng Zuo, Kai Du, Ao Li, Chen-Yu Zhang, Ren Guo, Ping Chen, Wei-Shuai Du, Yong-Li Zuo, Shu-Ming Li

**Affiliations:** 1 Graduate School, Beijing University of Chinese Medicine, Beijing, China; 2 Department of Pain Medicine, Beijing Hospital of Traditional Chinese Medicine, Capital Medical University, Beijing, China

**Keywords:** osteoarthritis, social media, quality of health information, TikTok, Bilibili, health communication, patient education

## Abstract

**Background:**

The proliferation of short video platforms has transformed public health communication, yet the quality of medical information shared on these platforms remains inconsistent. Osteoarthritis (OA), a prevalent and burdensome chronic condition, is frequently featured in online health content. However, the reliability of such information has not been systematically evaluated across major Chinese short video platforms. To assess and compare the quality and reliability of OA-related health information on TikTok and Bilibili, and to examine the influence of uploader type and user engagement metrics on content quality.

**Methods:**

In this cross-sectional study, a total of 189 OA-related videos were collected from TikTok (*n* = 96) and Bilibili (*n* = 93) using a standardized search strategy. Four validated instruments—the Journal of the American Medical Association (JAMA) benchmarks, modified DISCERN (mDISCERN), Global Quality Score (GQS), and Health on the Net Code (HONcode)—were used for video assessment. Each video was independently rated by two trained reviewers. Differences in quality scores were compared across platforms and uploader types (health professionals vs. non-professionals). Spearman correlation analysis was conducted to explore associations between video quality and engagement metrics (likes, comments, shares, favorites).

**Results:**

TikTok videos exhibited significantly higher median scores on JAMA (2.4 vs. 2.1, *P* = 0.001), GQS (3.0 vs. 3.0, *P* = 0.006), and HONcode (11.0 vs. 9.3, *P* = 0.005) compared to Bilibili. No significant difference was observed for mDISCERN scores. Videos uploaded by healthcare professionals had significantly higher GQS (*P* = 0.004) and HONcode scores (*P* = 0.010) than those from non-professionals. User engagement metrics were positively correlated with content quality, particularly on TikTok (e.g., likes vs. JAMA, *r* = 0.732, *P* < 0.001).

**Conclusions:**

OA-related videos on TikTok demonstrate higher overall quality and reliability compared to Bilibili, especially when created by healthcare professionals. User engagement metrics are positively associated with information quality, underscoring the importance of expert-led digital health communication. These findings highlight the need for platform-level interventions to promote trustworthy content and improve the digital health information ecosystem.

## 1 Introduction

Osteoarthritis (OA) is among the most prevalent chronic joint diseases worldwide, with its incidence steadily increasing due to global population aging (1). It primarily affects the knee, hip, and shoulder joints, significantly impairing mobility and reducing quality of life in older adults (2). The prolonged disease course and recurrent symptoms contribute to a substantial burden on both individual health and public healthcare systems (3, 4). Epidemiological studies demonstrate considerable regional variation in OA prevalence, with higher rates observed among older populations in developed countries (5). Beyond its direct physical impact, OA is associated with increased healthcare utilization and psychological distress (6, 7). Although available treatments—including pharmacological therapy, physical rehabilitation, and surgical interventions—offer symptom relief, their effectiveness varies, and some carry risks of adverse effects (8). Therefore, early screening, lifestyle modification, and enhanced patient self-management are critical components in OA care (9, 10).Research indicates that digital education has already played a role in osteoarthritis treatment, and some online education even outperforms patient self-management (11, 12).As OA requires long-term self-management, reliable online health information is particularly important for patient education.

The proliferation of internet access has transformed health information-seeking behaviors, with online platforms increasingly serving as primary sources of medical knowledge (13–15). Short video platforms such as TikTok and Bilibili have rapidly emerged as popular mediums for disseminating health information, owing to their intuitive, engaging, and easily shareable formats. Content on these platforms encompasses disease education, treatment options, patient experiences, expert commentary, and lifestyle guidance, offering opportunities for public health promotion (16).

Nevertheless, the quality and reliability of health information available on social media platforms vary widely (17–19). Algorithm-driven recommendation systems, favoring content with high engagement metrics, may inadvertently amplify low-quality or misleading medical information (20–22).Given the potential influence of such content on patient decision-making and health behaviors (23), evaluating the accuracy, credibility, and educational value of OA-related videos is imperative. Prior studies have assessed similar issues for other diseases (20, 24), but OA-related content on Chinese short video platforms remains underexplored.

Accordingly, this study aimed to systematically assess the quality and reliability of OA-related health information disseminated via TikTok and Bilibili. Specifically, we sought to (1) compare content quality between platforms, (2) examine the impact of uploader background (health professionals vs. laypersons) on video quality, and (3) investigate the relationship between content quality and user engagement metrics. To achieve this, we employed multiple validated evaluation instruments, including the Journal of the American Medical Association (JAMA) benchmarks, the modified DISCERN (mDISCERN) scale, the Global Quality Score (GQS) (25–27), and the Health On the Net Foundation's Code of Conduct (HONcode) (28).

## 2 Methods

This cross-sectional study aimed to assess the quality and reliability of OA-related health information disseminated via two major short video platforms, TikTok and Bilibili. A systematic search strategy, standardized video characterization, validated quality assessment instruments, and rigorous statistical analyses were employed to ensure comprehensive and objective evaluation.

### 2.1 Ethical considerations

This study analyzed publicly available video content from TikTok and Bilibili without collecting personal data or interacting with users. According to institutional and international guidelines, the study did not constitute human subjects research. Nonetheless, the protocol was reviewed and approved by the Institutional Review Board of Beijing Hospital of Traditional Chinese Medicine. The authors confirm compliance with TikTok's Terms of Use and Bilibili's platform policies.

### 2.2 Search strategy

To ensure the breadth and representativeness of the video sample, TikTok and Bilibili were selected as the primary data sources based on their large user bases and popularity in disseminating health-related content in China.

A new user account was registered on each platform to minimize the influence of personalized recommendation algorithms. The keyword “Osteoarthritis” (in Chinese: “骨关节炎”) was used to search for relevant videos. Searches were conducted in February 2025, without geographic restrictions, and included publicly available videos only. No filters were applied to sort the search results, thereby preserving the platforms' default ranking algorithms.

Although the study focused on the top-ranked search results to reflect real user exposure, the potential effect of algorithmic recommendation bias is acknowledged as a limitation.

### 2.3 Screening criteria

Videos were eligible if they met the following criteria: (1) explicitly related to osteoarthritis health education; (2) publicly accessible without privacy restrictions; and (3) duration between 0 and 60 min. Exclusion criteria included: (1) videos primarily addressing other diseases or pediatric populations; (2) advertisements unrelated to OA; and (3) duplicate videos or content lacking substantive OA relevance. An initial pool of 200 videos (100 from TikTok and 100 from Bilibili) was retrieved. After screening based on the predefined criteria, 189 videos were included in the final analysis, comprising 96 videos from TikTok and 93 videos from Bilibili. All inclusion and exclusion decisions were independently cross-checked by two reviewers to ensure consistency and transparency.

### 2.4 Video characterization

For each included video, a standardized set of characteristics was recorded, including basic information (video duration, main topic, and uploader identity), uploader type (categorized as healthcare professionals, such as osteopaths and rehabilitation therapists, or non-professionals, such as patients and health bloggers), content type (classified into disease knowledge, treatment methods, or lifestyle interventions, allowing for multiple categorizations per video), and user engagement metrics (number of likes, comments, favorites, and shares).

### 2.5 Video quality and reliability assessments

To systematically evaluate video quality, four validated instruments were employed: the Journal of the American Medical Association (JAMA) benchmarks, the modified DISCERN (mDISCERN) scale, the Global Quality Score (GQS), and the Health On the Net Foundation's Code of Conduct (HONcode).

The Journal of the American Medical Association (JAMA) benchmarks assessed the reliability of information sources and disclosure of conflicts of interest, with scores ranging from 0 to 4, where higher scores indicated greater credibility (Table 1) (20). The modified DISCERN (mDISCERN) scale evaluated clarity, reliability, balance, citation of external sources, and acknowledgment of uncertainties, using a binary scoring system (0 or 1 per item, total score 0–5) (Table 2) (29). The Global Quality Score (GQS) provided an overall assessment of educational value, rated from 1 (poor) to 5 (excellent) (Table 3) (25). The Health On the Net Foundation Code of Conduct (HONcode) assessed adherence to eight ethical principles for online health information, with a cumulative score ranging from 0 to 16 (Table 4) (30).

**Table 1:** Description of the JAMA benchmark criteria (4-point scale). Each criterion is scored as 1 if met, 0 if not. Total scores range from 0 to 4.

Criterion	Description	Score
Authorship	The identity of the video creator is clearly presented, including name, credentials, and institutional affiliation	0 or 1
Attribution	All sources of information, including references, data, and guidelines, are properly cited	0 or 1
Disclosure	Any sponsorship, advertising, funding, or potential conflicts of interest are clearly disclosed	0 or 1
Currency	The date of the content's creation and/or most recent update is clearly indicated	0 or 1

**Table 2:** Modified DISCERN (mDISCERN) scale criteria (5-point scale) used to assess the quality of OA-related video content.

Criterion	Description	Score
Clear Aims	Are the aims of the video clearly stated and achieved?	0 or 1
Reliable Sources	Is the information provided from reliable sources or expert authorship?	0 or 1
Reliable Sources	Is the information presented in a fair and objective manner?	0 or 1
Additional Resources	Are further references or resources provided for more information?	0 or 1
Additional Resources	Are areas of uncertainty or lack of consensus mentioned?	0 or 1

**Table 3:** Description of the Global Quality Score (GQS) used to evaluate the overall quality and patient usefulness of OA-related video content.

Quality level	Description	Score
Poor	Very poor quality, poor flow, significant information missing or misleading	1
Generally Poor	Generally poor quality, flow issues, some information missing.	2
Moderate	Moderate quality, acceptable flow, reasonably comprehensive information	3
Good	Good quality, clear flow, mostly comprehensive and valuable information	4
Excellent	Excellent quality, excellent flow, very comprehensive, accurate, high educational value	5

**Table 4:** Description of the health on the Net (HONcode) criteria used to evaluate the ethical quality and transparency of OA-related video content.

Principle	Criteria Description	Score
Authority	The qualifications of the author(s) or video creator are clearly stated	0–2
Complementarity	The content supports, not replaces, the patient-health professional relationship	0–2
Privacy	The video respects the confidentiality of user or patient data, if applicable	0–2
Attribution	Cited sources, publication dates, and references are provided.	0–2
Justifiability	Claims about benefits, effectiveness, or outcomes are evidence-based and justified	0–2
Transparency	Contact information or means of feedback are clearly available	0–2
Financial Disclosure	Funding sources, commercial support, or sponsorship are disclosed	0–2
Advertising Policy	Advertising content is clearly identified and separated from informational content	0–2

Video evaluations were performed independently by four trained experts following a randomized allocation of videos. A computer-generated randomization sequence assigned 189 videos into two groups of approximately equal size. Each group was evaluated by two independent reviewers (Group I: Shuming Li and Ren Guo; Group II: Ping Chen and Chenyu Zhang), who were blinded to the video source and uploader identity. In cases of substantial discrepancy between raters, a third independent clinical expert adjudicated the scores, with the final rating determined by the median value of the three evaluations. Prior to assessment, all reviewers underwent calibration training based on standardized scoring guidelines to ensure consistency and minimize subjective bias.

### 2.6 Statistical analysis

Continuous variables were presented as mean ± standard deviation (SD) or median and interquartile range (IQR), depending on the distribution. Categorical variables were expressed as frequencies and percentages. The Shapiro–Wilk test was used to assess the normality of continuous variables. Independent sample *t*-tests or Mann–Whitney *U* tests were applied to compare video quality scores (JAMA, mDISCERN, GQS, and HONcode) between platforms and uploader types. Spearman's rank correlation analysis was conducted to evaluate the associations between video quality scores and user engagement metrics. A two-sided *P*-value < 0.05 was considered statistically significant. All analyses were performed using SPSS version 27.0 (IBM Corp, Armonk, NY, USA).All analyses were independently verified by two statisticians to ensure accuracy and reproducibility.

## 3 Results

### 3.1 Video characterization

**Figure 1:**
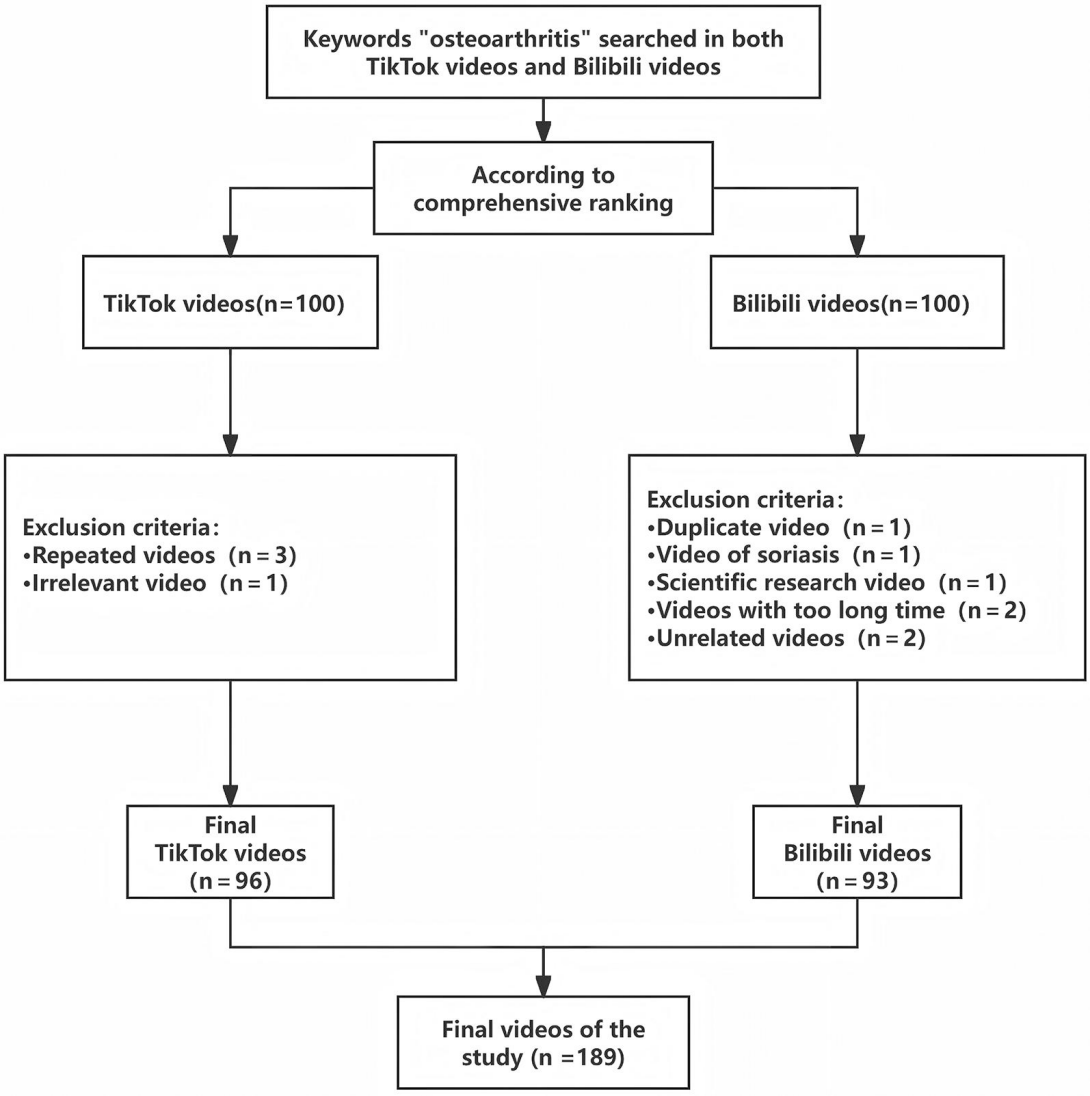
Flowchart of the overall study design.

A total of 200 osteoarthritis-related videos were initially retrieved, with 100 from TikTok and 100 from Bilibili. After applying predefined inclusion and exclusion criteria, 189 videos were included in the final analysis, comprising 96 TikTok and 93 Bilibili videos (Figure 1). Among these, 6 videos (6.3%) from TikTok and 28 videos (30.1%) from Bilibili were uploaded by non-professionals.

**Figure 2:**
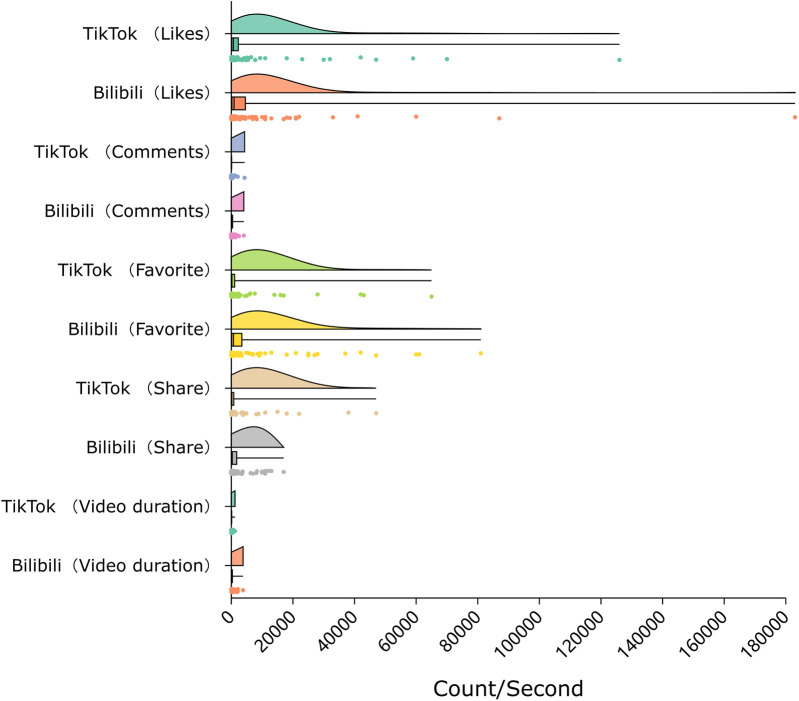
Distribution of video characteristics on TikTok and Bilibili. Violin plots show engagement metrics (likes, comments, favorites, shares; measured in counts) and video duration (measured in seconds).

Figure 2 summarizes the basic characteristics of the included videos. TikTok videos had a median duration of 75.5 s (IQR 49–108), compared to 152 s (IQR 72.5–332.5) on Bilibili (*P* < .001). The number of comments was significantly higher on Bilibili (median 128; IQR 15.5–424) than on TikTok (median 27; IQR 4–108.5; *P* = .002), as were favorites (median 697 vs. 179.5; *P* = .001). No statistically significant differences were observed in the number of likes (median 898 vs. 662.5; *P* = .351) or shares (median 297 vs. 148; *P* = .085) between the two platforms. The number of followers of each uploader was also recorded to provide additional context for engagement differences, although this variable was not included in inferential analyses.

### 3.2 Video quality scoring

**Figure 3:**
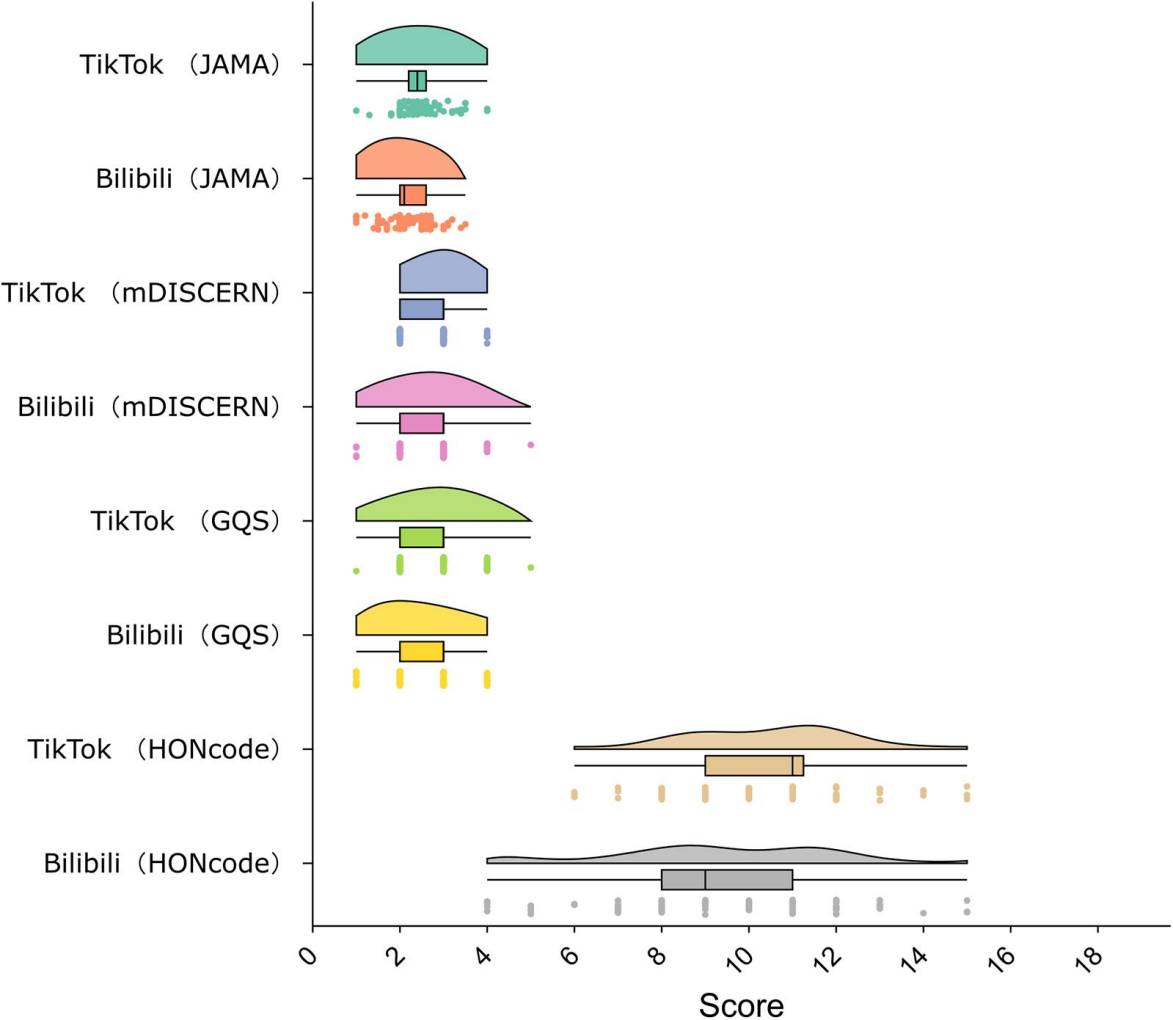
Distribution of video quality scores on tikTok and bilibili. Violin plots show results from four evaluation tools: JAMA (0–4), mDISCERN (0–5), Global Quality Score (1–5), and HONcode (0–16).

Video quality was assessed using four established scoring tools (Figure 3). TikTok videos had significantly higher JAMA scores (median 2.4; IQR 2.2–2.6) than Bilibili videos (median 2.1; IQR 2.0–2.6; *P* = .001). GQS scores were also higher on TikTok (median 3; IQR 2–3) compared to Bilibili (median 3; IQR 2–3; *P* = .006). HONcode scores were higher for TikTok (median 11; IQR 9–11.75) than for Bilibili (mean 9.33 ± 2.59; *P* = .005). No statistically significant difference was found in mDISCERN scores (median 3; IQR 2–3 for both platforms; *P* = .196).

### 3.3 Correlation analysis

**Figure 4:**
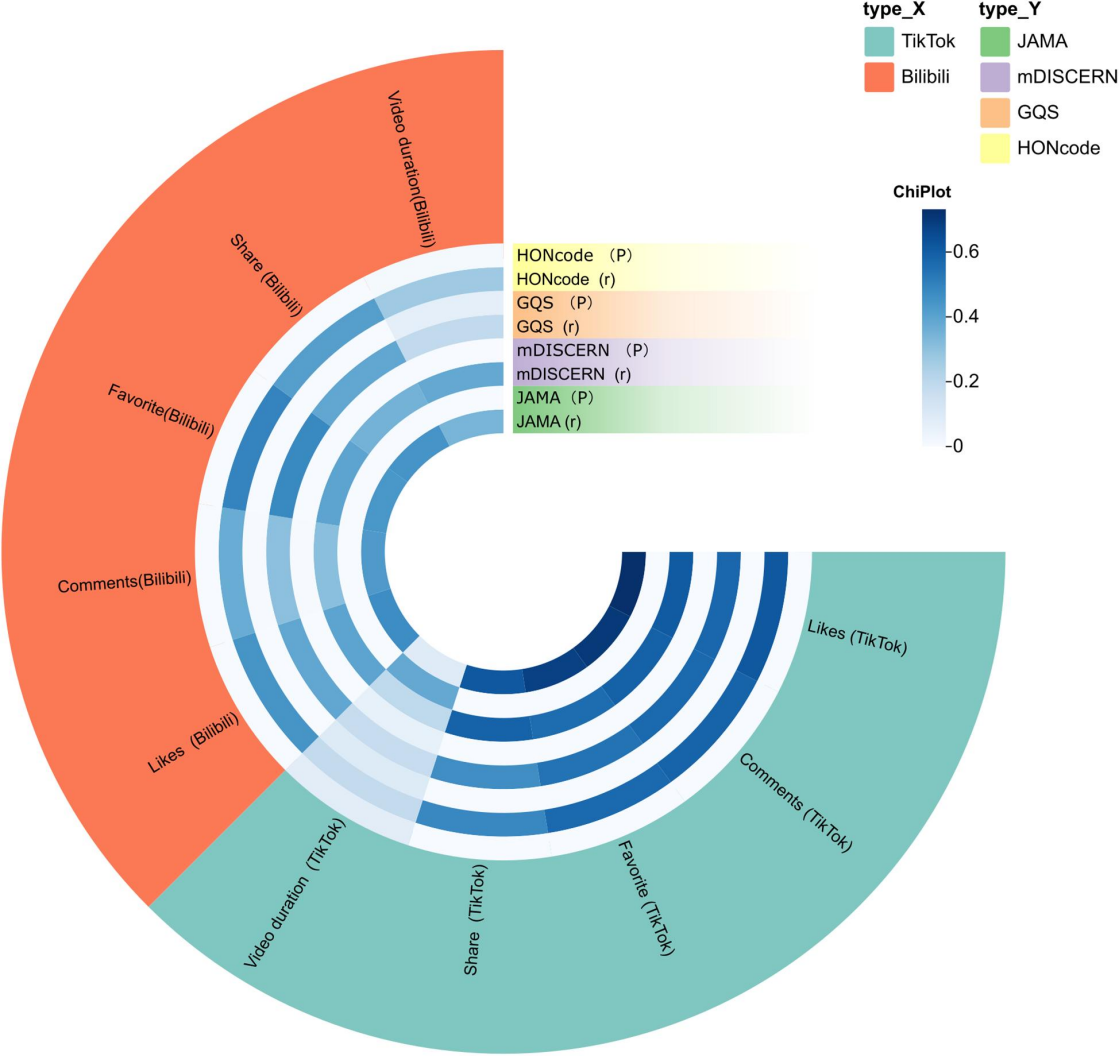
Correlation between user engagement metrics and video quality scores on tikTok and bilibili. Presented as a ring plot, outer segments represent engagement metrics, and inner rings display Spearman correlation coefficients (*r*) and *P* values for each quality score (JAMA, mDISCERN, GQS, HONcode).

Spearman correlation analysis was conducted to examine the associations between user engagement metrics and video quality scores on TikTok and Bilibili. Results are presented in Figure 4.

On TikTok, all engagement metrics were significantly positively correlated with JAMA scores, with the highest correlation observed for likes (*r* = 0.732, *P* < .001), followed by comments (*r* = 0.704), favorites (*r* = 0.684), and shares (*r* = 0.617). mDISCERN scores were also positively correlated with likes (*r* = 0.611), comments (*r* = 0.592), favorites (*r* = 0.561), and shares (*r* = 0.590; all *P* < .001). For GQS, correlation coefficients ranged from r = 0.467 (shares) to r = 0.578 (likes), and for HONcode from *r* = 0.483 (shares) to *r* = 0.618 (likes). Video duration was not significantly correlated with any score (*P* > .05).

On Bilibili, likes were moderately correlated with JAMA (*r* = 0.470, *P* < .001), mDISCERN (*r* = 0.399), GQS (*r* = 0.390), and HONcode (*r* = 0.449). Similar correlations were found for favorites (e.g., *r* = 0.480 for GQS; *r* = 0.495 for HONcode) and shares (*r* = 0.414 for HONcode). Comments also showed positive but weaker correlations with most scores (*r* = 0.309–0.430). Unlike TikTok, video duration was significantly correlated with JAMA (*r* = 0.344, *P* = .001), mDISCERN (*r* = 0.382, *P* < .001), and HONcode (*r* = 0.266, *P* = .010), but not with GQS (*P* = .071).

### 3.4 Comparison between groups

**Figure 5:**
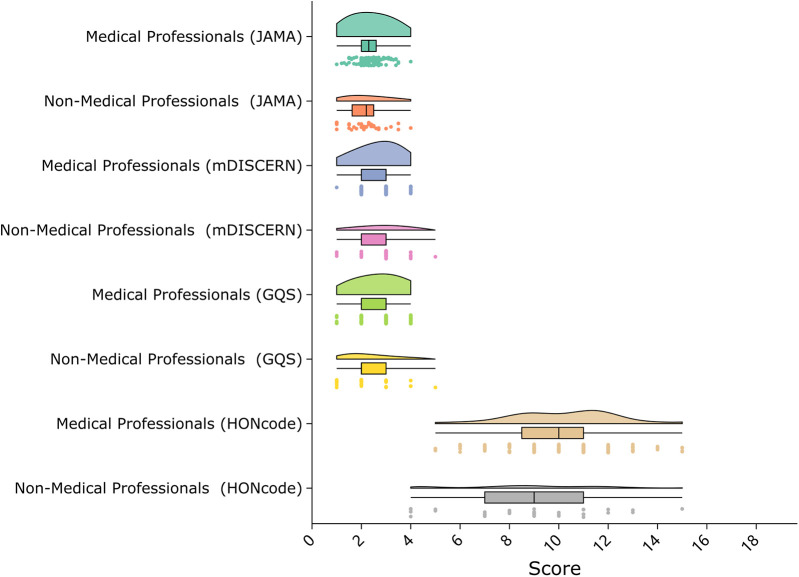
Distribution of video quality scores by uploader type. Violin plots show scores from four evaluation tools—JAMA (0–4), mDISCERN (0–5), Global Quality Score (1–5), and HONcode (0–16)—for videos uploaded by medical and non-medical professionals.

Comparison by uploader type revealed that videos from healthcare professionals had significantly higher GQS scores (median 3; IQR 2–3) compared to those from non-professionals (median 2; IQR 2–3; *P* = .004; Figure 5). HONcode scores were also significantly higher for professional uploaders (median 10; IQR 8–11) than for non-professionals (mean 8.76 ± 2.82; *P* = .010). No significant differences were found in JAMA scores (median 2.3; IQR 2.0–2.6 vs. mean 2.16 ± 0.76; *P* = .067) or mDISCERN scores (median 3; IQR 2–3 for both groups; *P* = .473).Subgroup analysis by content theme (disease knowledge, treatment, lifestyle) suggested a similar trend, though differences were not statistically significant.

## 4 Discussion

### 4.1 Platform differences and potential mechanisms

This study systematically evaluated the quality and reliability of OA-related health information on TikTok and Bilibili, revealing significant differences between the platforms. TikTok videos demonstrated higher quality across JAMA, GQS, and HONcode assessments, while mDISCERN scores showed no statistically significant difference. These findings suggest that, overall, TikTok may provide more credible and educational OA-related content than Bilibili. These results indicate an association rather than a causal relationship, given the cross-sectional naturse of the data.

Several factors may explain this disparity. First, platform algorithms likely influence the visibility of high-quality content (31). TikTok's algorithm is hypothesized to favor professionally produced, high-engagement videos, aligning with prior evidence showing that platforms such as YouTube prioritize trustworthy health information (25). In contrast, Bilibili's more decentralized and community-driven recommendation system may offer less curation, allowing content of variable quality to proliferate.

Second, differences in user demographics and video formats may shape content quality. TikTok's short-form, attention-driven design incentivizes concise, information-dense videos, while Bilibili's long-form culture may foster verbose or diluted messaging. Additionally, TikTok's recent moderation efforts targeting health misinformation may have elevated the baseline quality of content on the platform, while Bilibili continues to host a larger proportion of videos created by non-professionals, as reflected in our sample.

Importantly, while TikTok outperformed Bilibili in overall quality metrics, it remains susceptible to health misinformation (32). Prior research has demonstrated the presence of inaccurate or misleading videos even on well-regulated platforms. For example, in a study of sinusitis-related TikTok content, the majority of non-medical influencer videos contained inaccurate claims (33).This underscores the persistent need for quality assurance, regardless of platform. This highlights that algorithmic advantages do not necessarily eliminate misinformation risks, underscoring the need for ongoing monitoring and quality regulation.

### 4.2 Comparison with existing studies

Our results are consistent with previous studies demonstrating that healthcare professionals produce higher-quality content than non-professionals (34). Liang et al. similarly found that TikTok outperformed Bilibili in content reliability and educational value for videos about gastroesophageal reflux disease (20). This cross-condition consistency suggests structural platform advantages. However, such consistency should be interpreted as an observed pattern rather than a causal conclusion about platform mechanisms.

The observed superiority of professional content also echoes findings from YouTube-based studies, where professional authors achieved significantly higher GQS and mDISCERN scores than lay creators (25, 35).These results reinforce the importance of content source credibility in health communication.

Nonetheless, platform performance can vary by disease topic and cultural context. One study found that Chinese-language videos on Bilibili and TikTok outperformed English-language content on YouTube for gastric cancer, suggesting that public familiarity and topic sensitivity may influence content quality (24).

Regarding the relationship between content quality and user interaction, prior research has been mixed. Some studies have reported no significant correlation between engagement metrics and quality, while our findings demonstrate a strong positive association, particularly on TikTok. This may reflect algorithmic designs that amplify credible content, or user behavior that favors well-produced and informative videos (36, 37). Nevertheless, these correlations cannot confirm directional causality, as engagement may also drive algorithmic exposure or creator adaptation over time. In addition, We did not perform comment coding in this study, Further work may classify interaction types (e.g., medically relevant vs. casual comments) to assess “effective engagement” and its link to video quality.

### 4.3 Clinical and public health significance

This study underscores the critical role that short video platforms can play in patient education and chronic disease management. For individuals with osteoarthritis, access to accurate, high-quality online content can support informed decision-making, improve adherence to self-care practices, and potentially enhance long-term outcomes. The higher prevalence of reliable, professionally produced videos on TikTok suggests that certain platforms may offer more trustworthy health information environments than others (38). Nevertheless, these findings reflect associations observed within the current dataset and should not be generalized as an inherent superiority of one platform over another, since audience demographics and algorithmic mechanisms differ.

Given the chronic and self-managed nature of osteoarthritis, continuous access to concise and evidence-based content—particularly outside clinical settings—is essential. Videos produced by healthcare professionals may be especially effective due to their clarity, scientific accuracy, and ability to foster trust. Such content can serve as an extension of clinical care, reinforcing key messages and promoting sustained patient engagement (39, 40).

From a public health perspective, these findings suggest that social media platforms should be integrated into broader health communication strategies (41). To maximize public health benefits, platform operators should consider prioritizing evidence-based content in recommendation systems, introducing visible quality indicators (e.g., verification badges), and implementing mechanisms to flag or demote misleading material. Enhancing the discoverability of credible content—particularly from verified medical professionals—can improve the informational environment and reduce exposure to misinformation (42).

Furthermore, the observed association between video quality and user engagement supports the potential for high-quality content to achieve broader reach and impact. Public health agencies and healthcare institutions may benefit from partnering with digital creators or investing in content development to amplify accurate messaging at scale.

Finally, this study highlights the dual responsibility in digital health education: clinicians and public health professionals must actively engage in content dissemination while also equipping patients with critical appraisal skills to navigate an increasingly complex information landscape. Sustained improvement in online health information quality will thus require coordinated efforts among platforms, professionals, and users to balance accessibility with accuracy.

## 5 Limitations

This study has several limitations. First, the sample size of 189 OA-related videos may not fully capture the breadth and variability of content on TikTok and Bilibili. Future studies should consider larger and more diverse samples to improve generalizability. Second, the focus on a single disease—osteoarthritis—limits the applicability of findings to other medical domains. Expanding the scope to include additional health topics could yield a more comprehensive understanding of platform dynamics.

Third, although we employed four validated assessment tools (JAMA, mDISCERN, GQS, HONcode), the evaluation of video quality involves inherent subjectivity and does not directly assess clinical accuracy. Fourth, data access limitations precluded analysis of certain engagement metrics, such as view duration or algorithmic amplification, which may influence content visibility and user behavior. These constraints mean that platform-level mechanisms, such as algorithmic ranking and exposure bias, could not be fully captured, which may partly account for observed variations in engagement patterns. Finally, the cross-sectional design reflects a single time point, limiting the ability to assess temporal changes or causality. Therefore, the observed associations should be interpreted as descriptive rather than explanatory of underlying mechanisms.

Future research should incorporate longitudinal analyses, multi-language and multi-platform comparisons (43), and explore the effectiveness of content moderation strategies such as health labeling, verification badges, and user education initiatives to strengthen digital health literacy and content quality.Collaborative efforts among researchers, health authorities, and platform developers could further enhance the governance and transparency of online health information ecosystems.

## 6 Conclusion

This cross-sectional analysis revealed substantial variability in the quality and reliability of osteoarthritis-related health information across two major Chinese short video platforms. Overall, content on TikTok demonstrated higher credibility and educational value than that on Bilibili, particularly when produced by healthcare professionals. These observations reflect associations within the studied sample and should not be interpreted as evidence of inherent platform superiority. Importantly, video quality was positively associated with user engagement metrics, suggesting that well-crafted, accurate health content may also achieve greater visibility and influence. This correlation highlights the opportunity—but not the certainty—for credible content to reach wider audiences under current algorithmic dynamics. These findings underscore the critical role of platform governance and professional participation in shaping the digital health information environment. Future efforts should prioritize the integration of quality assurance mechanisms and the amplification of expert-led content to support informed health decision-making in the era of social media. By providing comparative data within the Chinese digital health context, this study contributes a localized perspective that can inform future international research on short-video health communication. Future research may also consider incorporating review mechanisms and algorithmic logic as mediating factors to better elucidate the pathways through which platform governance influences content quality and user engagement.

## Data Availability

The raw data supporting the conclusions of this article will be made available by the authors, without undue reservation.
